# The new normal of remote work: exploring individual and organizational factors affecting work-related outcomes and well-being in academia

**DOI:** 10.3389/fpsyg.2024.1340094

**Published:** 2024-02-12

**Authors:** Vincenza Capone, Giovanni Schettino, Leda Marino, Carla Camerlingo, Alessandro Smith, Marco Depolo

**Affiliations:** ^1^Department of Humanities, University of Naples Federico II, Naples, Italy; ^2^Area Organizzazione e Sviluppo, University of Naples Federico II, Naples, Italy; ^3^Ufficio Organizzazione e Performance, University of Naples Federico II, Naples, Italy; ^4^Alma Mater Studiorum – University of Bologna, Bologna, Italy

**Keywords:** post-pandemic, emerging psychosocial risks, psychosocial resources, university staff, well-being, remote working

## Abstract

**Background:**

Flexible work arrangements have become increasingly popular, driven by the widespread adoption of digital technologies in the workplace because of the pandemic. However, there is a scarcity of studies concerning remote work, especially related to technical-administrative staff (TAS) in academia. Therefore, the current study, adopting the Job Demands-Resources model, aimed to investigate the relationships between remote working self-efficacy, organizational support, techno-complexity, mental well-being, and job performance among TAS during remote working.

**Methods:**

A total of 373 individuals from TAS of a large Italian university participated in this study by completing a self-report questionnaire.

**Results:**

The findings showed positive and significant relationships between remote self-efficacy and job satisfaction as well as between such a perceived efficacy and mental well-being. Perceived support from supervisors acted as a protective factor against techno-complexity. In contrast, perceived support from colleagues emerged as able to promote well-being and job satisfaction. In addition, the latter was positively associated with well-being. Finally, individual job performance was positively affected by job satisfaction and negatively by techno-complexity.

**Conclusion:**

This study highlights the need for interventions to support TAS in remote working environments by leveraging employees’ self-efficacy as a key factor in reducing stress related to new technologies as well as enhancing well-being, job satisfaction, and, in turn, their performance.

## 1 Introduction

Flexible work practices, such as hybrid models combining remote work and in-person work, have gained popularity thanks to the recent pandemic, which accelerated the adoption of ICT (Information and Communication Technologies) in the workplace. In recent years, remote working has more than doubled in the Italian context: from 570.000 workers in 2019 to 1.8 million in 2020 (Eurofound and the International Labour Office, 2020; Observatory for Smart Working Italy, 2022). More broadly, public agencies have had to adapt their working environments to the unexpected challenges concerning COVID-19 (Athanasiadou and Theriou, 2021; Palumbo et al., 2023), fuelling a reconfiguration of work already underway and putting the accent on the ability of public sector organizations to manage human resources effectively (Boselie et al., 2021). More in detail, over the past decade, the public sector has undergone several changes, moving from a stable and predictable environment to one steeped in complexity and uncertainty (Pyun and Edey Gamassou, 2018), exacerbated by the pandemic. This has also led to a significant transformation in the content and structure of work (Brown, 1997) since they were often inadequate. This unprecedented event has impacted the balancing process between the demands of the organization and the resources available for individuals to address these new challenges effectively. The workload, particularly associated with technologies, has led to stress and, in turn, undermined organizational effectiveness and employees’ well-being (Pace et al., 2021; Nguyen and Tuan, 2022). Morea et al. (2023) showed that these professionals were able to redefine space, time, organizational involvement, and a better work-life balance during the pandemic. Nevertheless, during this time, the widespread use of remote work in the public sector raised concerns about the impact on workers’ well-being (Marino and Capone, 2021). These included excessive connectivity (where performance is evaluated more in terms of productivity rather than time spent), isolation from colleagues, and increased workload, as Bonacini et al. (2021) documented.

Scholars interested in the subject have highlighted the need to investigate how remote working has led to enduring changes in the work organization (Vyas et al., 2022). Following this aim, Todisco et al. (2023) conducted a study to examine the experiences of public sector employees with remote working, reporting that, after the pandemic, remote working significantly enhanced organizational flexibility and adaptability. However, issues still emerged regarding the right to disconnect and maintaining a healthy work-life balance.

Recent works showed similar findings by documenting that the rise in new technologies and changing organizational processes can be regarded as primary psychosocial risk factors in the working context (Sahut and Lissillour, 2023). The above-mentioned results can be explained by considering that remote work may make it more difficult to disconnect from work. On the other hand, remote work can also be plagued by distractions and interruptions, which are less likely to be experienced in a traditional office setting. Finally, isolation and limited social contacts linked to remote working can blur boundaries between work-related and personal activities to compensate for the lack of social ties (Prodanova and Kocarev, 2021).

Despite the relevance of this phenomenon, a paucity of studies has explored the experience of technical-administrative staff (TAS) with remote working (Burke and Pignata, 2020). To address this gap, we adopted the Job Demands-Resources (JD-R) model (Bakker et al., 2014) as the theoretical framework for this study, aiming to investigate the conditions of this professional population in remote working during the post-pandemic period. This perspective suggests considering the interaction between individual and organizational factors affecting work-related outcomes and individuals’ well-being. The following sections provide a description of the constructs evaluated in the research. These constructs, according to the JD-R model, were conceptualized as personal and organizational resources and demands, with potential outcomes related to job satisfaction, well-being and work performance.

### 1.1 Well-being and job satisfaction in remote working

Employees’ well-being in the workplace has emerged as a relevant issue for researchers (Shamsi et al., 2021), aiming to better understand its relationships with performance and to ensure adequate working conditions. Literature in this field (Medina-Garrido et al., 2017) suggests that employees who experience higher levels of well-being tend to report better job performance. Indeed, well-being factors, such as positive emotions, optimism, and resilience, can enhance individuals’ ability to perform well in their job (Bakker et al., 2014). It is well-established that *self-efficacy* beliefs can enhance performance across various domains (Bandura, 1986, 2001), including the working one. This reasoning is consistent with Mathis and Brown’s (2008) study reporting that work-related self-efficacy positively predicted successful performance and higher levels of job satisfaction, an indicator of work-related well-being.

In this regard, it is worth noting that there are different ways of conceptualizing well-being, both within and outside work settings. Warr (1994) suggested that well-being at work incorporates the construct of job satisfaction. This factor can determine a spillover effect influencing contexts outside of the work, as proved by prior studies confirming significant associations between job satisfaction and satisfaction with life (Tadić et al., 2013) as well as the different dimensions of well-being (Capone and Petrillo, 2020). The latters contribute to a multifaceted well-being definition, aligning with Keyes’s (2007) perspective, which regards it as a comprehensive state of emotional, psychological, and social health. In this vein, an individual experiencing well-being not only lacks psychopathological disorders but also enjoys positive emotions and shows effective functioning across diverse life domains (Westerhof and Keyes, 2010), including job. The two dimensions of occupational and mental well-being are conceptually different aspects (Wright and Cropanzano, 2000) that should be explored further in today’s working world: as some recent studies point out (Tapas, 2022), an exploration of the relationship of the antecedents of these two variables is the first step in understanding the relationship between them.

Although the literature on remote working has not extensively adopted this perspective (Burke and Pignata, 2020), prior studies documented that job-related factors such as individual abilities, task complexity, and organizational support can play a pivotal role in employees’ well-being. For instance, Di Tecco et al. (2021) examined work engagement and job satisfaction in flexible work settings, concluding that clarity in their professional role and the absence of conflicting roles positively affect individuals’ work engagement and job satisfaction. Moreover, supervisor and peer support emerged as a key factor in increasing workers’ work engagement. The latter, in addition, has been identified as an antecedent of job performance (Rana et al., 2019).

Finally, when employees enjoy higher levels of flexibility in doing their job, they tend to report increased job satisfaction and, in turn, better well-being (Mohammed et al., 2022). In this regard, it can be argued that individuals who can tailor their job according to their needs – for example, by leveraging remote working features – are more able to balance work and life duties and, as such, experience improved job satisfaction (Hanglberger and Merz, 2011; Schall, 2019) and well-being (Charalampous et al., 2019).

Given the above, we expected that performance was positively predicted by job satisfaction and well-being. In addition, we expected that job satisfaction positively predicted well-being.

### 1.2 Examining remote working demands and resources through the JD-R model

In the Work and Organizational Psychology literature, the JD-R model has been widely adopted to investigate factors impacting individual and organizational outcomes (Zeike et al., 2019). More in detail, Bakker et al. (2007) focused on work-related variables defined as *job demands* and *job resources*. The formers are conceived as “physical, psychological, social, or organizational aspects of the job that require sustained physical and/or psychological (cognitive and emotional) efforts or skills” (Van den Broeck et al., 2013, p. 85). The latters are regarded as “physical, psychological, social, or organizational aspects of the job that are either/or functional in achieving work goals, reducing job demands and the associated physiological and psychological costs, or in stimulating personal growth, learning, and development” (p. 85).

#### 1.2.1 Technostress as an outcome of remote working challenges

Technostress and its relationships with remote working have been largely investigated (Oh and Park, 2016; Spagnoli et al., 2020) since it constitutes a pivotal stress manifestation in the context of digitalization processes about work activities. Literature has defined this construct as “a modern disease of adaptation” (Bondanini et al., 2020, p. 2), “the amount of stress that a person experiences and manifests when using a specific type of technology, or when he/she is in direct or indirect contact with it” (Castillo et al., 2020, p. 18).

A central dimension of technostress is *techno-complexity* (Schettino et al., 2022a,b), which occurs when employees perceive their skills are inadequate due to the difficulties associated with adopting new technologies required in their job (Tarafdar et al., 2015; Molino et al., 2020). In this vein, a lack of computer skills can lead employees to believe they cannot manage the complexity of technologies, requiring them to spend more time understanding how to use these technologies. With this regard, it is well-acknowledged that techno-complexity is higher among older individuals (Marchiori et al., 2019) and that Italian TAS in academia has an average age of about 52 years (ANVUR, 2023). Taken together, these considerations led us to focus on this dimension of technostress to evaluate which factors could affect such a kind of stress among Italian TAS. Regarding the organizational factors, in a prior study by Mudrak et al. (2018) on academic staff, the findings proved that job resources, such as employees’ control over work and support from colleagues/supervisors, were positively associated with work engagement and job satisfaction. Conversely, job demands, including job insecurity and work–family conflicts, were found to be positively related to experienced stress. These processes emerged relatively independent, suggesting that academics can remain committed to their work despite increased demands, especially when provided with adequate labor resources. The authors argued that flexible work arrangements can benefit staff as they can help reduce stress. A subsequent study conducted in the post-pandemic period (Mondo et al., 2023) examined the relationships between workload during remote work and technostress among Italian public administration employees who were working remotely. The results showed that techno-invasion and techno-complexity played as mediators between workload and well-being.

Thus, we expected that techno-complexity was a risk factor for well-being, job satisfaction and performance.

#### 1.2.2 Self-efficacy and remote working

Self-efficacy is a factor that aids employees in effectively handling challenging job demands and bolsters their confidence in achieving their goals. In the JD-R model, self-efficacy is defined as a personal resource, a “belief in one’s capabilities to organize and execute the courses of action required to produce given attainments” (Bandura, 1997, p. 3). It can act as a mediator between job resources and engagement/exhaustion by affecting perceived job resources (Xanthopoulou et al., 2007). Therefore employees with high self-efficacy exhibit greater confidence, autonomy, and positive psychological outcomes than those with lower self-efficacy (Hackman and Oldham, 1975; Capone et al., 2021). This belief is shaped by repeated skilled experiences that influence one’s perceptions of capability and task challenges (Bandura, 1986) as well as lower stress and technostress levels (Bandura, 2001; Capone et al., 2021; Yener et al., 2021), resulting in higher performance (Caprara et al., 2004). Therefore, it can be argued that work self-efficacy plays a buffer role against the detrimental impact of technostress on employees by reducing emotional exhaustion (Ma et al., 2021). As Bandura (1997) posited, efficacy beliefs can improve performances in different contexts. However, in order to enhance its predictiveness of individual behaviors, it is necessary to consider specific manifestations of such a belief (Bandura, 2000; Capone and Petrillo, 2020). In this vein, work self-efficacy proved its predictiveness of employees’ performance (Stajkovic and Luthans, 1998). Following this line of reasoning, literature on remote working (Prodanova and Kocarev, 2021) has shown a strong association between the self-efficacy of individuals working remotely and the dimensions of technostress (Bahamondes-Rosado et al., 2023).

Consequently, we expected that remote working self-efficacy positively affected job satisfaction, well-being, and performance. In addition, we expected that job satisfaction and well-being positively affected performance. Lastly, we expected that techno-complexity was negatively influenced by remote working self-efficacy.

#### 1.2.3 The role of organizational support in remote working

Social support is one of the possible resources that can help individuals in coping with stress (Hobfoll, 2001). In the work context, it manifests as *organizational support*, an essential job resource able to counteract job strain (Bakker et al., 2007). It is conceptualized as a global construct (House et al., 1988), emerging from multiple sources, including supervisors and colleagues (Kossek et al., 2011).

The literature has highlighted several positive outcomes associated with adequate organizational support. Academic staff who perceive strong organizational support tend to experience higher job satisfaction, increased engagement, commitment and performance. Conversely, when academic staff perceive a lack of support, they may form turnover intentions and experience a reduction in their job effectiveness (Mazzetti et al., 2023).

It must be underlined that organizational support plays a pivotal role across various hierarchical levels. In support of such a thesis, Armstrong-Stassen (1998) documented that managers with higher perceived support reported greater job satisfaction than those with less support. This result may be understood by taking into account that support can enhance individuals’ trust in the organization and the belief that their efforts will be recognized and rewarded. In the academic context, a study by Yaghi and Bates (2020) highlighted that organizational support from supervisors and peers improved the individuals’ ability to transfer content learned in training into daily practices.

Furthermore, supervisor support can help academic staff by providing them with career advancement opportunities and feedback (Yang et al., 2018). The latter, in addition, is an antecedent of adequate levels of self-efficacy (Zhang and Wang, 2021) because it can promote a positive and healthy work environment through listening to concerns and helping members cope with the challenges associated with their roles. Consequently, effective supervisor support contributes to employees’ satisfaction with work and better performance (Bak, 2020; Mazzetti et al., 2023). At the same time, peer support emerged as effective in enhancing collaboration, motivation, and job satisfaction. More in detail, when they perceive to be emotionally supported in their work, they are less likely to experience negative outcomes related to their well-being (Jawahar et al., 2007; Felstead and Henseke, 2017; Charalampous et al., 2019). Specifically, peer support emerged as a factor affecting the relationship between technostress and well-being (Timms et al., 2015; Di Tecco et al., 2021).

Net of the benefits associated with different manifestations of social support in the workplace, as stated by Cohen and Wills (1985), people who perceive high levels of support are more inclined to evaluate situations as less stressful since they perceive stressors as more manageable and less threatening. In other words, social support acts as a “buffer” against detrimental outcomes: employees who perceive support from their supervisors and colleagues tend to improve their psychological resources to deal with work-related stress and, in turn, experience higher well-being (Westman and Chen, 2017; Demerouti and Bakker, 2023). In other words, social support can reinforce workers’ coping skills, allowing them to better deal with work-related challenges (e.g., adopting remote working practices) and relative stressors improving, thereby, job satisfaction and well-being. This thesis aligns with the literature on technostress, highlighting the role of social support in mitigating the impact of technostress on employees’ well-being and performance (e.g., Weinert and El-Robrini, 2021).

In line with the above, we expected peer and supervisor support to affect job satisfaction and well-being positively. In addition, we expected that techno-complexity was negatively affected by these kinds of support.

## 2 The current study

Although prior studies (Burke and Pignata, 2020) have explored factors promoting academic well-being using the JD-R model (Demerouti and Bakker, 2023), a literature gap remains regarding the relationships between remote working and mental well-being in post-pandemic scenarios. In addition, while the literature (Yener et al., 2021) has investigated the relationships between self-efficacy and technology-related stress, to our knowledge, no previous study has considered the role of self-efficacy and techno-complexity in the context of remote working for Italian TAS, during the post-pandemic era. In the current study, we focused on a group of workers from a public administration. These professionals have begun to adopt remote working largely during the COVID-19 pandemic; however, the transition from “emergency” to “structural” flexible work practices necessitates research to effectively enhance working conditions and practices. Thus, addressing the aforementioned gap in the literature, we conducted the following study and discussed the potential and practical implications of our results.

**Figure 1:**
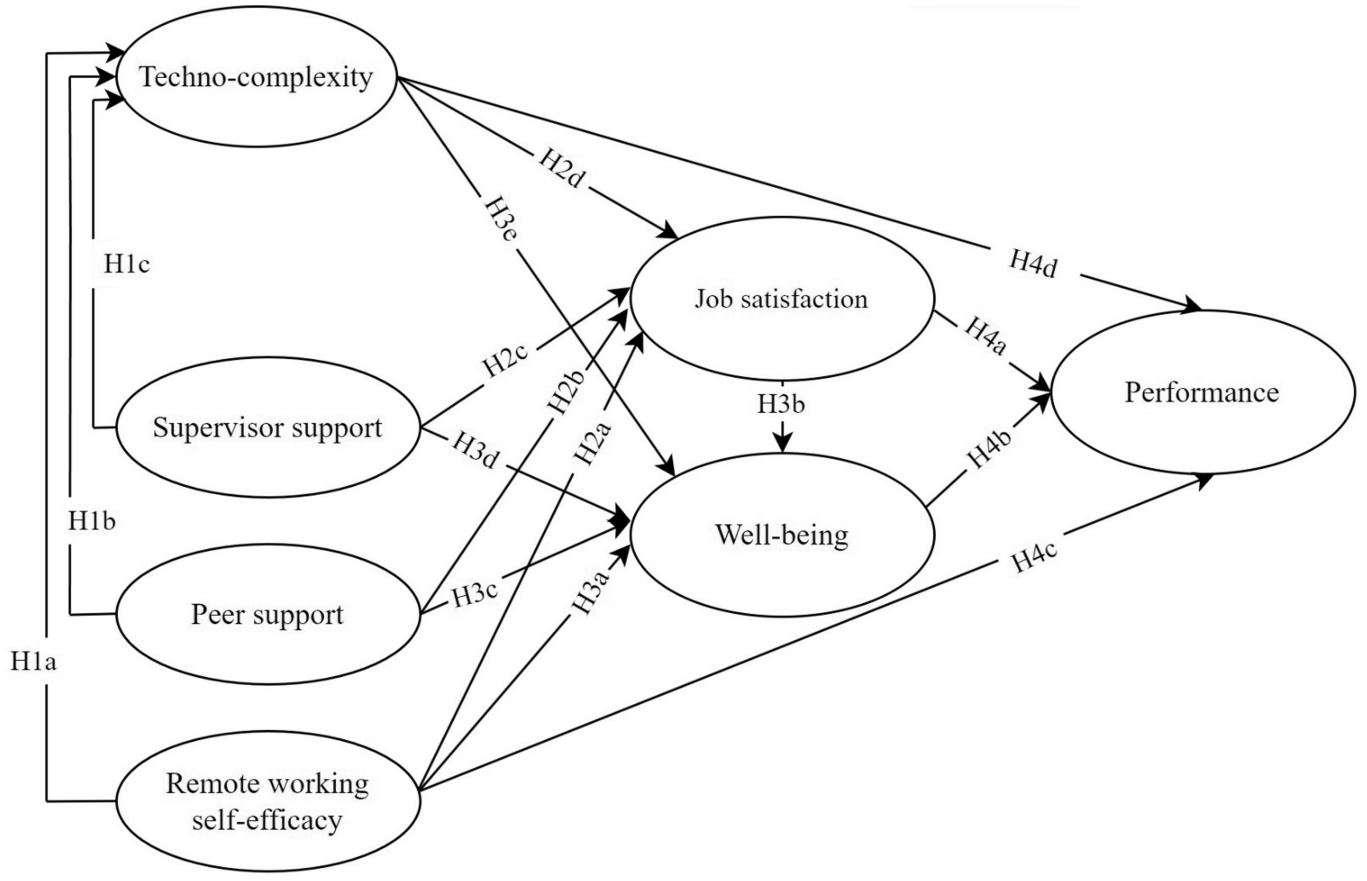
Hypothesized model.

More in detail, we hypothesized as follows (Figure 1):

Hypothesis 1 (*H1*). Techno-complexity was negatively predicted by remote working self-efficacy (Hypothesis *H1*a), peer (Hypothesis *H1*b), and supervisor support (Hypothesis *H1*c).

Hypothesis 2 (*H2*). Job satisfaction was positively predicted by remote working self-efficacy (Hypothesis *H2*a), peer support (Hypothesis *H2*b), supervisor support (Hypothesis *H2*c), and, negatively, by techno-complexity (Hypothesis *H2*d).

Hypothesis 3 (*H3*). Well-being was positively predicted by remote working self-efficacy (Hypothesis *H3*a), job satisfaction (Hypothesis *H3*b), peer support (Hypothesis *H3*c), supervisor support (Hypothesis *H3*d) and, negatively, by techno-complexity (Hypothesis *H3*e).

Hypothesis 4 (*H4*). Performance was positively predicted by job satisfaction (Hypothesis *H4*a), well-being (Hypothesis *H4*b), and remote working self-efficacy (Hypothesis *H4*c). Techno-complexity negatively predicted performance (Hypothesis *H4*d).

### 2.1 Procedure and participants

A convenience sample of TAS from a large Italian university participated in the study. Employers were invited through an e-mail to fill out a web-based self-report questionnaire of 15 min about their experience with remote working. The questionnaire was accessible only to the university employees by entering login data. In order to take part in this study, participants had to meet the following criteria: to be of legal age (1), (2) to be part of TAS (2), and to have worked in remote mode during the previous year (3). Before proceeding with the recruitment of participants, a pre-test was conducted to ascertain the usability and technical functionality of the questionnaire. Additionally, an *a priori* power analysis was performed for Structural Equation Models (Soper, 2022) to determine the adequate sample size based on the number of observed variables (20) and latent (7) in the hypothesized model. Specifically, considering a medium-sized effect (E.S. = 0.30), alpha = 0.05, and a power of 0.80, the results indicated that a minimum number of 170 participants would have been appropriate to obtain the specified effect, given the considered structure of the model.

A total of 373 individuals took part in the study by signing the informed consent form and completing the questionnaire. Therefore, the sample size seemed appropriate for verifying the statistical hypotheses. Participation was voluntary and anonymous; no incentive was given, and respondents were allowed to withdraw from the study at any time. Data were exclusively used for the purposes of this study and were accessible solely to the research team. All procedures followed were in accordance with the Helsinki Declaration (World Medical Association, 2013) and the General Data Protection Regulation. Data were collected between February and March 2022.

### 2.2 Variables and measures

In the first section of the questionnaire, participants filled out the informed consent form. Subsequently, they were instructed to answer all questions by thinking specifically about remote working. Then, the following measures were administered in the same order to all the participants.

#### 2.2.1 Remote working self-efficacy

The 6-item Work-Efficacy Scale (Borgogni et al., 2001) was used. The instrument measures workers’ beliefs in their ability to effectively manage various tasks, commitments, and challenges related to their professional role (6 items, e.g., “I am always able to master the emergencies and unexpected events related to my work”). Each item was rated on a 7-point scale ranging from “strongly disagree” (1) to “strongly agree” (7). The internal reliability of the scale in the current study was good (Cronbach’s Alpha = 0.86; McDonald’s Omega = 0.86).

#### 2.2.2 Peer support

The 4-item Colleagues’ support subscale of the Quality at Work Tool (AQ@workT; Brondino et al., 2022) was used. The Colleagues’ support subscale evaluates individuals’ perception of co-workers’ support (e.g., “Colleagues give me the help and support I need”). Participants were asked to indicate their degree of agreement on a 7-point Likert scale ranging from “totally disagree” (1) to “totally agree” (7). The internal reliability of the scale in the current study was good (Cronbach’s Alpha = 0.85; McDonald’s Omega = 0.93).

#### 2.2.3 Supervisor support

An item was used to measure the perceived supervisor support. Participants were asked: “How do you evaluate the support given by your supervisors?” The item was on a 6-point scale ranging from “poor” (1) to “excellent” (6).

#### 2.2.4 Techno-complexity

The 4-item Techno-complexity subscale of the Italian version (Molino et al., 2020) of the Technostress Creators Scale (Ragu-Nathan et al., 2008) was used. The Techno-complexity subscale evaluates workers’ perception of inadequacy due to the ICT features and complexity (e.g., “I do not know enough about technology to handle my job satisfactorily”). Participants were asked to indicate their degree of agreement on a 5-point Likert scale ranging from “strongly disagree” (1) to “strongly agree” (5). The internal reliability of the scale in the current study was good (Cronbach’s Alpha = 0.88; McDonald’s Omega = 0.88).

#### 2.2.5 Job-satisfaction

An item was used to measure the participants’ satisfaction regarding their job (i.e., “What is your level of satisfaction with your job?”) by following indications of Cortese and Quaglino (2006). The item is rated on a 7-point Likert scale ranging from “I am extremely dissatisfied” (1) to “I am extremely satisfied” (7).

#### 2.2.6 Well-being

Three items from the Mental Health Continuum-Short Form scale (MHC-SF; Petrillo et al., 2015) were used to measure individuals’ mental well-being. Participants answered the items “In the past month, how often did you feel … satisfied with life, that the way our society works makes sense to you, that you had experiences that challenged you to grow and become a better person” by using a 6-point Likert scale ranging from “never” (0) to “always” (5). The internal reliability of the scale in the current study was good (Cronbach’s alpha = 0.80; McDonald’s omega = 0.80).

#### 2.2.7 Performance

An item was used to measure participants’ perceived performance in remote work (i.e., “How did individual performance change in remote working time?”). The item is rated on a 4-point Likert scale ranging from “decreased” (1) to “improved” (4).

#### 2.2.8 Demographic information

The questionnaire included a socio-demographic section where participants were required to provide their age, marital status, education level, and professional role in the university.

### 2.3 Statistical analysis

Statistical analyses were conducted using the statistical software R version 4.3.2. In addition, descriptive statistics and reliability indices (Cronbach’s Alpha and McDonald’s Omega) were examined for all study variables. These indices are considered adequate when their values are ≥0.70 (Nunnally, 1978; Kalkbrenner, 2023). Moreover, Pearson’s correlations were calculated to evaluate the association among the variables. In order to test the hypothesized model, we carried out a full structural equation model (SEM; Jöreskog, 1970). Since our data were not completely normally distributed, Skewness and Kurtosis values > |1| for remote working self-efficacy, peer support, job satisfaction, and peer support, parameters were estimated using the maximum likelihood estimation with robust standard errors and a Satorra-Bentler scaled test statistic (“MLM” estimator in R package lavaan; Rosseel, 2012). As Maydeu-Olivares (2017) suggested, MLM allows obtaining the goodness-of-fit statistics in situations where normality assumptions are violated. This involves calculating standard errors and a mean-adjusted chi-square test statistics that are robust to non-normality. Then, we estimated the hypothesized structural relationships. The goodness of fit was evaluated using the following indices: Chi-square test (*χ*^2^), Comparative Fit Index (CFI), Tucker-Lewis Index (TLI), Root Mean Square Error of Approximation (RMSEA), and Standardized Root Mean Square Residual (SRMR). The fit can be considered adequate with a non-significant Chi-square, CFI and TLI values of at least 0.95, and RMSEA and SRMR values lower than 0.08 (Hu and Bentler, 1999). It must be underlined that the sample size influences the Chi-square test, which tends to be significant with large samples (Bentler and Bonett, 1980). For this reason, it is appropriate to look at the other fit indices. The scaling method adopted to assign a scale to every latent variable consisted of fixing their variance to 1 (Barbaranelli and Ingoglia, 2013). All the answers to the questionnaire were mandatory, so there were no missing values. Effects of the considered predictors on the main dependent variable (i.e., performance) were estimated by controlling for non-psychological variables that, according to preliminary analysis, showed a significant correlation with individuals’ performance. Specifically, participants’ age showed a positive relationship with their performance *r* = 0.14, *p* < 0.001.

## 3 Results

### 3.1 Sample characteristics

Most participants were women (62.2%), mainly aged between 50 and 60 years (*n* = 141). Regarding marital status, 72.7% were cohabiting or married, and 27.3% declared single. Regarding education level, most participants had a degree (66.8%) and were not serving as supervisors (83.4%).

### 3.2 Descriptive statistics

Concerning psychological variables (Table 1), the findings showed that, on average, participants reported a very high level of self-efficacy related to remote working (*M* = 6.13; *SD* = 0.80), quite high levels of job satisfaction (*M* = 5.68; *SD* = 1.34), performance (*M* = 2.99; *SD* = 0.99), and perceived peer (*M* = 5.50; *SD* = 1.26) and supervisor support (*M* = 5.30; *SD* = 0.90), moderate levels of well-being (*M* = 3.27; *SD* = 1.24). Moreover, despite the large number of participants aged between 50 and 60 years, the analyses reported low techno-complexity levels (*M* = 1.88; *SD* = 0.82). Concerning the correlations between the variables, it emerged as follows: self-efficacy, perceived peer support, job satisfaction, well-being, and performance positively correlated with each other. In addition, techno-complexity related to remote working was negatively associated with well-being and performance. Lastly, based on the analyses of the correlations among the variables, we excluded serious multicollinearity concerns, as no correlation between the independent variables of the hypothesized model was >0.80 (Kline, 2005).

**Table 1:** Descriptive statistics and Pearson’s correlations among variables.

	Mean (SD)	1	2	3	4	5	6	7
1. Remote working self-efficacy	6.13 (0.80)	1						
2. Peer support	5.50 (1.26)	0.43^**^	1					
3. Supervisor support	5.30 (0.90)	0.30^**^	0.42^**^	1				
4. Techno-complexity	1.88 (0.82)	0.22^**^	−0.08	−0.13^*^	1			
5. Job satisfaction	5.68 (1.34)	0.30^**^	0.33^**^	0.35^**^	−0.09	1		
6. Well-being	3.27 (1.24)	0.29^**^	0.27^**^	0.27^**^	−0.14^**^	0.26^**^	1	
7. Performance	2.99 (0.99)	0.21^**^	0.16^**^	0.04	−0.16^**^	0.24^**^	0.19^**^	1

^*^p < 0.05; ^**^p < 0.01. SD = standard deviation.

### 3.3 Structural equation model results

**Figure 2:**
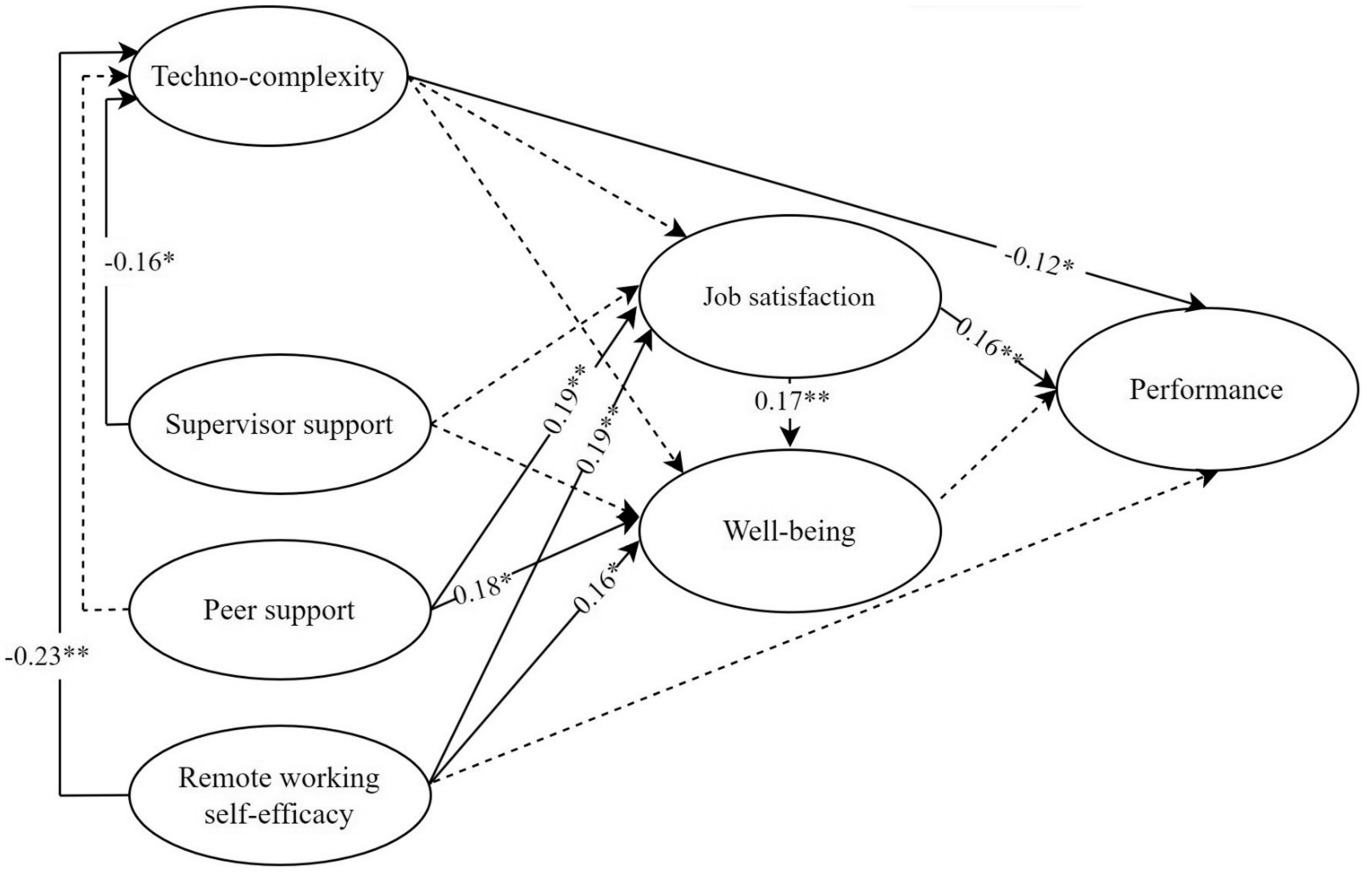
Structural equation model results. ^*^p < 0.05; ^∗∗^*p* < 0.01. The dashed lines indicate not significant paths.

The findings indicated that, with the exception of the Chi-square (*χ*^2^ = 316.250, *df* = 170, *p* < 0.001), the model (Figure 2) provided a satisfactory fit to the data, with CFI = 0.950; TLI = 0.935; RMSEA = 0.056; SRMR = 0.053. The evaluation of the degree of freedom supports the consideration of the identifiability of the model, which explained 16% of the variance in job satisfaction, 17% in well-being, and 11% of the variance in performance. Results showed that almost all the hypotheses were confirmed. Concerning the measurement model, all factor loadings were statistically significant (*p* < 0.001), indicating that all items contributed to measuring the related constructs. Regarding the structural model, the findings showed that remote working self-efficacy (*β* = −0.23, *p* < 0.01) and supervisor support (*β* = −0.16, *p* = 0.03) negatively predicted techno-complexity, confirming *H1*a and *H1*c. Nevertheless, contrary to what was hypothesized (*H1*b), peer support did not significantly affect techno-complexity (*β* = 0.12, *p* = 0.07). However, such a support was positively associated with job satisfaction (*β* = 0.19, *p* < 0.01) and well-being (*β* = 0.18, *p* < 0.05), confirming *H2*b and *H3*c. In addition, well-being was not significantly associated with techno-complexity (*β* = −0.09, *p* = 0.16) and supervisor support (*β* = −0.00, *p* = 0.97), in contrast with H3e and *H3*d. However, consistent with our hypotheses (*H3*a, *H3*b), employees’ well-being was positively predicted both by self-efficacy (*β* = 0.16, *p* = 0.03) and job satisfaction (*β* = 0.17, *p* = 0.01). Finally, in line with the hypotheses, techno-complexity (*H4*d; *β* = −0.12, *p* = 0.03), job satisfaction (*H4*a; *β* = 0.16, *p* < 0.01), were significantly associated with performance (control variable effect on performance: age: *β* = −0.09, *p* = 0.07), whereas remote self-efficacy (*H4*c; *β* = 0.11, *p* = 0.06) and well-being (*H4*b; *β* = 0.11, *p* = 0.05) were not.

## 4 Discussion and conclusion

Universities have undergone, and continue to undergo, internal changes that endanger the work-life balance of their employees. The recent pandemic has brought about structural changes in work, including a reorganization of processes and a sudden and massive recourse to technology. These elements have been identified as major psychosocial risk factors for lecturers and TAS. The pandemic has also given a strong impetus to remote work, in many cases without a planning/transition phase, or in any case by accelerating processes that were only in their initial stages. After the pandemic, the rise of technologies (e.g., smartphones, virtual meetings) has allowed alternative work arrangements, which have provided workers with enhanced flexibility in their job. However, this phenomenon has also led to unclear boundaries between individuals’ work and private life.

Hence, despite the positive evaluation of remote working, a growing number of researchers have also begun to highlight some negative aspects of it (Riva et al., 2021). In particular, one of the most significant risks is that of nurturing an “always-on” culture, with an unmanageable extension of work commitments (Dagnino et al., 2020), with detrimental consequences consisting of work-related fatigue and exhaustion (Rinaldi and Riyanto, 2021). With many universities reporting the use of remote working as a structured practice, it is important, as well as urgent, to identify what should be done to improve the implementation of these new ways of working both at an organizational and individual level. For these reasons, our research aimed to investigate the role of self-efficacy, techno-complexity and organizational support in remote working experience among TAS in Italy, primarily focusing on their well-being and job performance during the post-pandemic era.

Using the JD-R model as the theoretical framework for our study (Bakker et al., 2014), we have considered the techno-complexity as a new organizational demand (Molino et al., 2020) that can shape mental and job well-being (Pace et al., 2021), while remote working self-efficacy and organizational support as resources.

The results largely confirmed our hypotheses. In line with studies by Bandura (1997, 2000, 2001), self-efficacy emerged as a protective factor against techno-complexity and a promoter of both occupational and mental well-being. Self-efficacy is a specific belief that could, therefore, enable employees to mitigate the stressors linked with technologies, including those adopted with remote working and, in turn, to work sustainably and healthily from any location (Grant and Clarke, 2020). Similarly, our results are aligned with evidence from the literature highlighting that perceived social support is negatively associated with work-related stress (Macías et al., 2019). However, it is worth noting that not all perceived support has the same protective effect: after all, social support could act as an effective buffer when it is responsive to the demands arising from stressful situations (Azpíroz-Dorronsoro et al., 2023). The results of our work emphasize that supervisor support can act as a resource to counteract stress related to technologies in remote working, while peer support can promote job satisfaction and mental well-being. In this vein, these results highlight that techno-complexity is not only a consequence of job demands but also depends on the personal relationships in the workplace.

Besides, our results suggest that scholars and practitioners when referring to well-being, should make a clear distinction between “well-being at work” and “context-free well-being,” with the aim of improving both of them. Moreover, the study highlights the crucial role of job satisfaction in employees’ performance, strengthening the literature on the topic that reports ambiguous results (Bowling, 2007). The findings also support the thesis, assuming that the relationships with job-related antecedents are stronger for job-related well-being. As such, our study could provide a more comprehensive insight into how specific organizational factors can shape well-being.

Furthermore, these findings are part of a larger stream of studies that emphasize how important it is to analyze the antecedents of job satisfaction and to distinguish between factors that promote it and those that hinder it (Williams et al., 2023). Careless management of the work environment, especially with regard to negative emotions, can certainly create quite a few problems for job management, satisfaction, and ultimately performance.

Finally, in line with Keyes (2007) suggestion, it is important to investigate both well-being and malaise because the presence of one does not exclude the absence of the other. Although negatively correlated, techno-complexity had no significant relationship with well-being. At the same time, the former was related to performance. Hence, subsequent studies should better explore this relationship.

### 4.1 Limitations

It is essential to acknowledge some limitations of the present study.

First, sharing the invitation to complete the questionnaire via e-mail might have yielded self-selection bias. In particular, it is plausible to suppose that most participants were those who were already inclined towards remote working via a more frequent use of digital tools. Additionally, the findings are based on self-reported data, potentially subject to memory bias and respondents’ fatigue.

A further issue regards the distribution of the participants, which is not representative of the Italian academic TAS population. Therefore, future studies should also consider comparisons between different universities located in different parts of Italy in terms of both the characteristics perceived by the local community and the resources and services objectively available. Furthermore, the well-being of academic TAS is affected not only by work-related issues but also by non-academic factors, such as the socio-cultural, environmental and psychological circumstances they experience.

Moreover, while the implementation of a single-item approach (e.g., job satisfaction) has been demonstrated to be adequate for assessing certain constructs (Wanous et al., 1997; Nagy, 2002; Chang et al., 2022), such measures may not fully capture the multifaceted nature of the inherent variables. Consequently, this could limit the ability to generalize our results across diverse contexts or populations.

Finally, since the study adopted a cross-sectional design, the relationships described should be considered carefully and cannot allow for causality inferences. Therefore, future research should adopt a longitudinal approach to address such a limitation and provide a clearer understanding of causal dynamics among considered variables.

### 4.2 Practical implications

Our work is part of the strand of studies that aim to understand how the use of remote work may have accelerated the digitization process and the consequences for well-being and work performance. The preferred setting was universities, with a focus on TAS on whom the literature is lacking.

From a research perspective, our results contribute to strengthening the literature that supports the easy and versatile implementation of the JD-R model in different contexts and situations (Bakker and de Vries, 2021).

Furthermore, by including occupational and mental well-being, the study reinforces the importance of considering these two dimensions as significant but different, emphasizing the need to analyze and capture the relationship with performance as well (Diedericks and Rothmann, 2014).

In addition, our findings contribute to the literature about remote working by adding evidence from a population heavily affected by post-pandemic digital transformation, such as TAS in universities.

From the standpoint of suggestions for stakeholders affected by the ongoing digital change, the study provides several operational suggestions.

First of all, conceptual models like the JD-R (Demerouti and Bakker, 2023) reinforce the hypothesis that the most effective approach in interventions is a context-targeted one. What the JD-R model offers is proof of the existence of a series of stronger relationships between certain variables. However, the specific influence of a given variable (for instance, remote working self-efficacy, in our case) on the considered outcome may vary. The added value of the model- and the small contribution we tried to offer- in terms of practical implications, was a sort of check-list of factors (work-related or person-related) that an organization should carefully consider and contextualize, in order to strive to maximize the beneficial effects of all existing resources, and minimize the detrimental effects of all existing demands.

Literature seems to suggest that such a process is similar to what happens in human resource management (HRM): recent research (Lepak et al., 2004) suggests that the real impact on desired outcomes comes more from an HRM System and depends less on the quality of single actions (e.g., recruitment, selection, onboarding etc.). Hence, the suggestion could be to answer the question: “What can we do to increase the probability that our employees (depending on the specific working situation), develop a stronger perception of being able enough to cope with the job-related challenges?

Therefore, universities need to gain awareness of how the intensive use of ICT can affect their employees’ personal and professional well-being, as well as their performance. They also need to establish appropriate strategies to help them cope with job demands related to digital transformation, improving in such a way employees’ productivity and well-being. This study, showing protective factors and charting the path of personal and organizational resources to be strengthened, provides important practical implications in the field of human resource management.

The results emphasize the importance of organizational support and efficacy beliefs for workers’ well-being, highlighting the need for differentiated training (Schettino and Capone, 2022), also based on the requests of staff and the goals that the organization itself wants to pursue (e.g., improve stress management, enhance satisfaction). Hence, boosting individuals’ confidence in their capability is essential for increasing their overall well-being and improving the organization’s performance (Caprara et al., 2004). Of course, such a boosting strategy should not rely only on the spontaneous and personal engagement of employees but should be accompanied by specific and personalized supporting interventions on work goals and work skills to enhance effort and motivation: people confronted with a perceived “affordable” challenge are more likely to put effort on it.

Implementing effective development programs and their evaluation methods requires a more targeted approach, which entails considering factors such as social support as well as the perception of job satisfaction. This consideration means efforts to improve staff working conditions should be tailored to specific goals and values (Capone and Petrillo, 2020). Besides, when employees maintain positive relations with other organizational members, it can be improved the identification and mitigation of external and internal demands (Van Droogenbroeck et al., 2014). In addition, increased attention to specific support could also improve the overall effectiveness of human resource practices.

Finally, since techno-complexity can occur when individuals are forced to make efforts to understand how to use new technologies without the necessary skills, using new technologies in remote working needs acquiring new skills, which can significantly affect the relative perception of complexity in using them and, consequently, individuals’ well-being. As suggested by our findings, this can happen mainly when employees are not supported in the adoption of these new technologies, for example, by adequate training (Mondo et al., 2023; Schettino et al., 2024) that, thereby, should be implemented to avoid such a stress and improve employees’ well-being and performance.

The above-mentioned implications could be of great use in the post-pandemic era, especially in the context of a hybrid work model that blends remote work with in-office work, where the technological investments made can be leveraged.

## Data availability statement

The raw data supporting the conclusions of this article will be made available by the authors, without undue reservation.

## Ethics statement

Ethical review and approval was not required for the study on human participants in accordance with the local legislation and institutional requirements. The studies were conducted in accordance with the local legislation and institutional requirements. The participants provided their written informed consent to participate in this study.

## Author contributions

VC: Conceptualization, Data curation, Formal analysis, Investigation, Methodology, Project administration, Software, Supervision, Visualization, Writing – original draft, Writing – review & editing, Funding acquisition. GS: Conceptualization, Data curation, Formal analysis, Funding acquisition, Investigation, Methodology, Software, Validation, Visualization, Writing – original draft, Writing – review & editing. LM: Conceptualization, Data curation, Investigation, Validation, Visualization, Writing – original draft, Writing – review & editing. CC: Data curation, Investigation, Methodology, Project administration, Visualization, Writing – review & editing. AS: Software, Supervision, Validation, Visualization, Writing – review & editing. MD: Validation, Visualization, Writing – review & editing.
